# Cataract-Causing Defect of a Mutant γ-Crystallin Proceeds through an Aggregation Pathway Which Bypasses Recognition by the α-Crystallin Chaperone

**DOI:** 10.1371/journal.pone.0037256

**Published:** 2012-05-24

**Authors:** Kate L. Moreau, Jonathan A. King

**Affiliations:** Department of Biology, Massachusetts Institute of Technology, Cambridge, Massachusetts, United States of America; University of Arkansas for Medical Sciences, United States of America

## Abstract

**Background:**

The transparency of the eye lens depends upon maintenance of the native state of the γ- and β-crystallins, which is aided by the abundant chaperones αA- and αB-crystallin. Mature onset cataract, the leading cause of blindness worldwide, involves the polymerization of covalently damaged or partially unfolded crystallins into light-scattering aggregates. A number of single amino acid substitutions and truncations of γ-crystallins result in congenital cataract in both humans and mice, though in many cases the coupling between the protein alterations and the accumulation of aggregates is poorly defined.

**Methodology/Principal Findings:**

We have studied the aggregation properties and chaperone interactions of human γD-crystallin carrying substitutions of two buried core mutants, I90F and V75D, which cause congenital cataract in mice. The *in vitro* aggregation pathway competing with productive refolding was not altered by either substitution. Furthermore, this aggregation pathway for both mutant proteins–originating from a partially folded intermediate–was efficiently suppressed by αB-crystallin. Thus the cataract pathology was unlikely to be associated with a direct folding defect. The native state of wild-type human γD-crystallin exhibited no tendency to aggregate under physiological conditions. However both I90F and V75D native-like proteins exhibited slow (days) aggregation to high molecular weight aggregates under physiological conditions. The perturbed conformation of I90F was recognized and bound by both αA and αB chaperones. In contrast, the aggregation derived from the perturbed state of V75D was not suppressed by either chaperone, and the aggregating species were not bound by the chaperone.

**Conclusions/Significance:**

The cataract phenotype of I90F in mice may be due to premature saturation of the finite α- crystallin pool. The V75D aggregation pathway and its escape from chaperone surveillance and aggregation suppression can account for the congenital cataract pathology of this mutant. Failure of chaperone recognition may be an important source of pathology for many other protein folding defects.

## Introduction

Amino acid substitutions in diverse human proteins are associated with a variety of pathologies. In some cases, these reflect loss of the activity of the native state, for example, the G551D and G1349D substitutions of CFTR result in aberrant channel opening [1]. In other cases, such as the sickle cell mutation and hemoglobin polymerization, they induce a polymeric, though native-like state resulting in the pathology [2]. In yet other instances, substitutions may cause protein folding defects or increased off-pathway aggregation. Examples include mutations in transthyretin [3]–[5], lysozyme [6] and α_1_-antitrypsin [7], [8]. However, for many mutant proteins our understanding of how the substitution leads to the defect remains obscure. Given the large number of proteins that require interaction with various classes of chaperones, it seems likely that some defects classified as protein stability or protein folding defects may reflect a failure to be recognized by the appropriate chaperone. A well-characterized example is the tumor suppressor VHL. The wild-type (WT) protein is recognized by the group II chaperonin CCT; however, CCT recognition of oncogenic mutants is altered [9].

Human γD-crystallin (HγD) is one of the three major γ- crystallins required for transparency of the human lens. It is present in high concentrations in the lens nucleus, which is formed *in utero* during early development. The terminally differentiated lens fiber cells lack organelles including nuclei and ribosomes. Thus, proteins synthesized prior to differentiation must maintain their native structures and solubility over a lifetime. Cataract, the leading cause of blindness worldwide, arises from the aggregation of lens proteins resulting in opacification of the tissue.

While primarily a disease intimately linked with advanced age, numerous cases of hereditary and congenital cataract in both humans and mice are associated with mutations in the γ-crystallin genes [10]–[12]. The effects of a number of these amino acid substitutions on the properties of the γ-crystallins have been studied in detail. Surface replacements of arginine residues including R36S and R58H in HγD lowered the barrier to crystallization resulting in rare crystal cataracts [13], [14], rather than the aggregated state found in mature onset cataracts. The P23T HγD substitution altered solubility of the native state [15], [16]. Sandilands *et al* [17] showed that three different mouse crystallin variants resulted in aggregation of the mutant proteins within the lens fibers, and Moreau and King [18] reported destabilization of HγD by buried core substitutions. Zhang *et al* [19] found similar destabilization by the G61C substitution in HγD. In γS-crystallin, the G18V substitution significantly destabilized the native state [20]. However, studies of the properties of the mutant proteins under chemical or thermal stress do not account satisfactorily for the aggregation observed in cataractous lenses under physiological conditions.

A fuller elucidation of the molecular mechanism of crystallin aggregation is essential for understanding cataract formation and for the development of prophylactics. Kosinski-Collins and King used atomic force microscopy to image growing aggregates formed upon dilution of denatured HγD into buffer [21]. While these aggregates appeared fiber-like, they were not amyloid in nature [21]. Further study of this aggregation pathway revealed that the C-terminal domain (C-td) of HγD must be at least partially unfolded for aggregation to proceed [22]. Molecular dynamics simulations identified specific regions of the C-td that could serve as nuclei for aggregation [23]. Oxidative damage to susceptible side chains has been proposed as a major mechanism of protein destabilization within the lens, but the coupling to aggregation is not fully established [24]–[29]. Amyloid-like pathways have also been reported for damaged or acid-treated proteins [17], [30]–[32]. Recent evidence suggests that regions of the C-td of HγD are responsible for amyloid formation [33].

The passive chaperone α-crystallin is also present at high concentrations in the lens. Horwitz first showed that α-crystallin possessed a strong molecular chaperone activity by suppressing the thermal aggregation of the bovine β_L_- and γ-crystallin fractions of soluble lens protein [34]. It also suppressed the aggregation of a range of other proteins including insulin, α-lactalbumin, apolipoprotein C-II, citrate synthase, alcohol dehydrogenase and α-synuclein [34]–[40]. With respect to its physiological substrates in the lens, studies have demonstrated the ability of α-crystallin to suppress βγ-crystallin aggregation, including WT and deamidated βB2 [41], truncated βB1 [27], and the major γ-crystallins found in human lenses [22]. The chaperone may also suppress the aggregation of cytoskeletal proteins such as intermediate filaments [42], [43]. Several mutations in the α-crystallins are associated with cataract in mice and humans [10]. Though both αA and αB are present in the lens, only αA knockouts display a cataractous phenotype [44], [45].

As with many chaperones, α-crystallin does not bind the lens crystallins in their native states [22], [46], but appears to recognize regions of partially unfolded or covalently damaged chains. Both αA and αB bound destabilized mutant versions of β-crystallins when incubated together, and this binding was correlated with the population of an unfolding intermediate of mutant βB2-crystallin [47]. Cataracts are very rare in humans below the age of about 50, and this presumably reflects protection from protein aggregation by α-crystallin. However, the α-crystallin pool is likely saturated in older adults [48], so that the lens loses its capacity for chaperone protection from protein damage and unfolding.

Here we present analyses of the aggregation behavior of WT and mutant HγD proteins carrying the amino acid substitutions V75D and I90F. These mutants represent substitutions in the hydrophobic core of each double Greek Key domain. They are associated with congenital cataract in mice [11], [49] and were previously found to destabilize HγD [18]. Recent studies indicated that the double mutant I4F/V75D (known as I4F/V76D in [50]) sufficiently perturbed the protein conformation to enable α- crystallin binding in the absence of aggregation [50].

Unfortunately, terminally differentiated primary lens fiber cells cannot be maintained in cell culture, limiting studies to lens epithelial cells or other cell types, where γ-crystallins are not normally found at high levels. Therefore, *in vitro* experiments were performed to study two aggregation pathways. The first derives from a partially folded intermediate associated with productive refolding. It represents a model of misfolding that may occur during the initial translation and folding events within the lens. The second pathway results from a species derived from a destabilized native-like state *in the absence* of denaturant. This models destabilization and local or global unfolding that may occur over time after translation and productive folding of the mutant chains.

For some mutants, congenital cataract formation may represent the destabilization and subsequent misfolding of the γ- and β- crystallins [20]. In other cases, mutations lead to alterations in solubility while stability is maintained [13]–[16], [51]. The results reported here suggest that failure of α-crystallin to rescue altered crystallin chains from aggregation is a likely contributor to cataract for some congenital mutants, and may also contribute to mature onset cataract.

## Results

### Wild-type and Mutant HγD Protein Aggregation Compete with Refolding

Kosinski-Collins and King observed the aggregation of WT HγD when rapidly diluted from the unfolded state in 5.5 M guanidinium hydrochloride (GdnHCl) to buffer with residual denaturant concentrations below 1 M [21]. This aggregation pathway is in kinetic competition with the productive refolding pathway of the protein. The aggregating polypeptide chains–visualized by atomic force microscopy–first formed small globular assemblies and then filamentous structures [21]. Based on knowledge of the unfolding/refolding pathway of HγD, the aggregation-prone intermediate species appeared to have a fully unfolded N-terminal domain (N-td) and a partially unfolded or otherwise destabilized C-td [22]. In particular, it was shown that partial unfolding and population of the stable intermediate at 2.5 M GdnHCl did not result in appreciable aggregation upon dilution to 0.5 M GdnHCl [22]. Instead, higher initial GdnHCl concentrations were required. A similar prerequisite for aggregation was observed for the mutant proteins L5S, V75D and I90F HγD [18].

**Figure 1:**
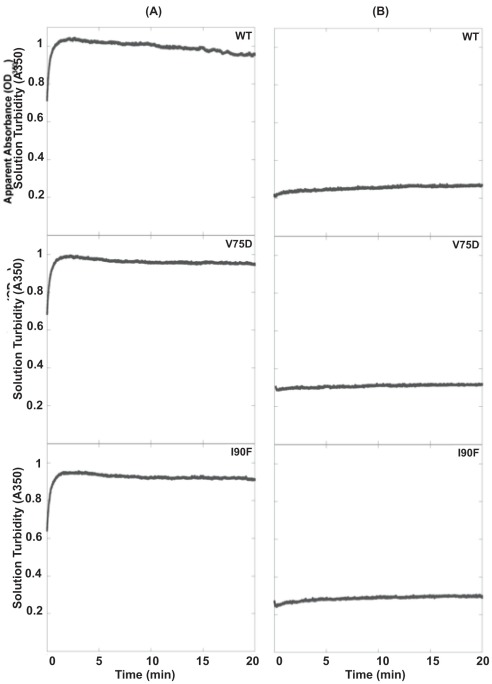
The aggregation of WT and mutant HγD in the absence and presence of αB. (A) Aggregation and (B) suppression reactions for WT, V75D, and I90F HγD. Solution turbidity was monitored for 20 minutes to follow the formation of light-scattering aggregates upon rapid dilution of HγD protein out of 5 M GdnHCl. For suppression reactions αB was present in a 5-fold molar excess in the dilution buffer. Each graph is labeled in its upper right corner with the protein name.

**Figure 2:**
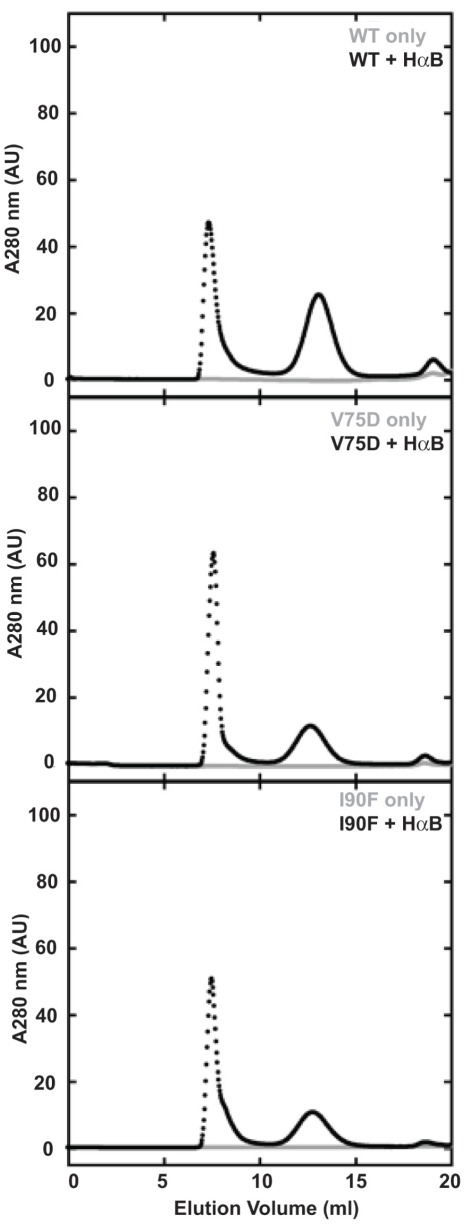
Size exclusion chromatograms of aggregation and suppression samples. Aggregation is shown in gray and aggregation suppression is shown in black, with the suppression samples containing a 5-fold excess of αB. Each chromatogram is labeled in its upper right corner with the protein name.

To evaluate the partitioning of protein between the aggregation and productive refolding pathways, we performed aggregation assays for WT, V75D and I90F HγD. All proteins were denatured in 5 M GdnHCl at 37°C and then diluted with buffer to initiate refolding and competing aggregation. Solution turbidity was monitored as the absorbance at 350 nm (A350) due to light scattering by the growing aggregates. Changes in A350 were essentially the same for WT and both mutant proteins (Figure 1A). The A350 increased very quickly over the first ∼2 minutes of the reaction before reaching a maximum of about 1 AU for both the WT and mutant proteins. The overall change in A350 was 0.31 for WT HγD, 0.36 for V75D, and 0.35 for I90F (Table 1). The aggregation reaction was kinetically favored over refolding of HγD to the native monomer. When chromatographed over a Superose 6 size exclusion chromatography (SEC) column, very little native protein was recovered. Notably, high molecular weight (HMW) species were absent at earlier elution volumes, indicating that the aggregated species were too large to pass through a 0.2 µm membrane filter and smaller oligomeric species were not present at detectable levels (Figure 2, gray traces). Thus, this assay, which monitors aggregation competing with refolding, did not detect significant differences between the WT and mutant crystallins.

**Table 1:** Solution Turbidity Measurements for WT and Mutant HγD in the Absence and Presence of αB.

Protein	ΔA^1,2^		Maximum A350^1^	
	− αB	+ αB	− αB	+ αB
WT	0.31±0.05	0.06±0.01	0.94±0.1	0.27±0.06
V75D	0.36±0.04	0.06±0.01	1.0±0.05	0.27±0.08
I90F	0.35±0.05	0.07±0.01	1.1±0.1	0.31±0.04

1 Units are Absorbance Units (AU) and means ± standard deviations are given.

2 ΔA  =  maximum A350 – minimum A350.

### Suppression of HγD Aggregation by αB

Acosta-Sampson and King studied the chaperone activity of αB against aggregation of three abundant human γ-crystallins and found that it differentially suppressed their aggregation [22]. Although the mutants discussed here appeared to aggregate through a similar pathway–attested to by their similar aggregation kinetics and overall levels of aggregation–it is possible that the passive chaperone αB could interact with one or both of the mutants in a different manner than with WT, resulting in a change in aggregation kinetics or overall suppression levels. To this end, aggregation suppression experiments were performed in the same manner as the previously described assay, with the addition of a 5- fold molar excess of αB in the refolding buffer.

Under these conditions, αB suppressed the aggregation of the WT and mutant proteins to similar extents (Figure 1B). The maximum A350 for both WT and V75D was 0.27 AU, while that of I90F was slightly higher at 0.31 AU. There was a slight increase in solution turbidity over the time course of the experiment and no initial burst in absorbance. The ΔA values were similar at ∼0.07 for all three proteins in the presence of the chaperone (Table 1). Based on the maximum A350 values, αB suppressed the aggregation of WT HγD by 72%. In comparison, V75D aggregation was suppressed by 73% and that of I90F by 70%.

Analysis of these suppression reactions by SEC resulted in the separation of two distinct peaks (Figure 2, black traces). The first peak eluted in the void volume and was composed of long-lived complexes of αB and HγD. The second major peak corresponded to excess chaperone and eluted in a broader peak around 13 ml. The native HγD peak was minor in the case of WT and negligible for both mutant proteins. These results are in agreement with previous observations that the void volume peak contains protein complexes [22]. As with the aggregation assay alone, the assay to measure suppression of aggregation did not differentiate the mutant proteins from the wild type.

### Interactions of αB with Initially Native HγD

The suppression assays described above, which investigated the *in vitro* refolding pathway and the competing aggregation pathway, were conducted with HγD initially unfolded in 5 M GdnHCl. Other aggregation pathways could originate from a native-like state that may fluctuate and unfold over time, populating conformations that may be aggregation-prone. Within the lens we would not expect an unfolding pathway from the native state to be the reverse of the folding pathway or aggregation pathway of newly synthesized nascent chains released from ribosomes.

To address this, purified WT, V75D and I90F HγD were incubated for 28 days at 37°C in the presence or absence of either αA or αB. SEC was performed at 0, 14, 21, and 28 days to determine whether soluble protein was lost to aggregation and/or if the chaperones formed complexes with the WT or mutant proteins. Samples were filtered before chromatography and large aggregates were not detected with this method. As controls, each protein, WT, V75D and I90F, was incubated individually, as were αA and αB.

**Figure 3:**
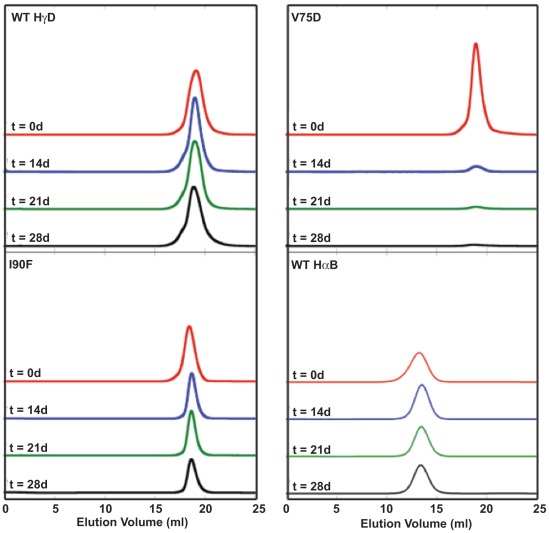
Size exclusion chromatograms of single protein controls for native mixing experiments. Separate samples were prepared for each time point in SEC buffer. In all cases proteins were present at 1 mg/ml. Each chromatogram is labeled in its upper left corner with the protein name.

In control samples without chaperone, the levels of both WT and mutant HγD proteins decreased over time, as shown by the decreased peak size in later chromatograms (Figure 3). However, the amplitude of the decrease was not the same across all proteins. WT HγD had the least change in monomeric protein levels over the course of the experiment. In the case of V75D, virtually no protein peak was visible by SEC after 14 days of incubation and white insoluble material was visible by eye. These aggregates could be dissolved with 2% SDS and a prominent band at 20 kDa was present upon SDS-PAGE analysis (see later Results). Smaller fragments were not detected by gel electrophoresis, further confirming that V75D was not proteolytically degraded during the incubation. The recovery of I90F was intermediate and large-scale aggregation was not observed as for V75D. αB was unaffected by the prolonged incubation, while the elution volume of αA was slightly earlier after incubation was complete (Figures 3 and 6, respectively).

**Figure 4:**
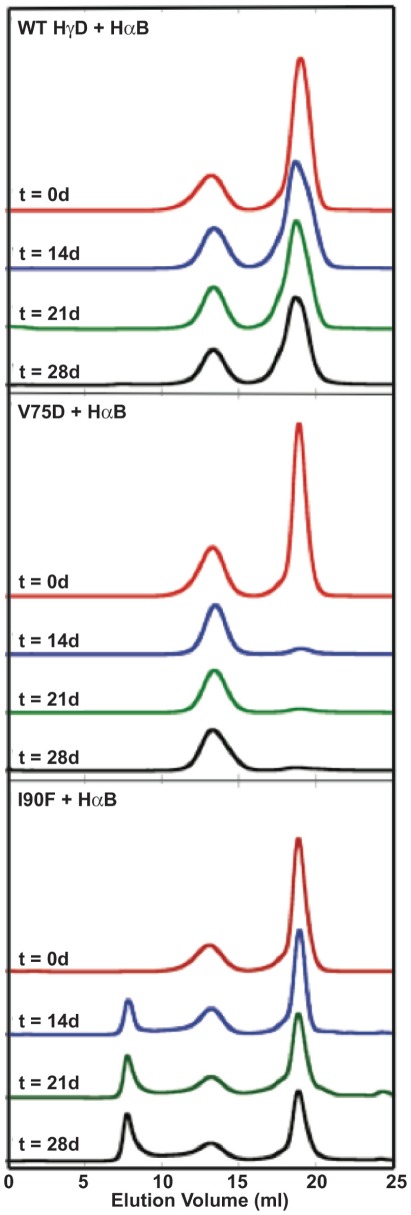
Size exclusion chromatograms of native protein mixtures containing WT or mutant HγD and αB chaperone. Separate samples were prepared for each time point in SEC buffer. Times given are in days. In all cases proteins were present at 1 mg/ml. Each chromatogram is labeled in its upper left corner with the protein mixture.

**Figure 5:**
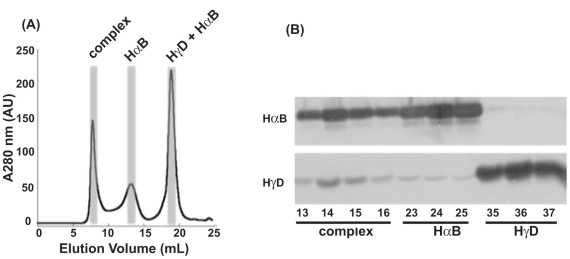
Western Blot analysis of I90F + αB native mixing. (**A**) Size exclusion chromatograms of the I90F + αB mixture after a 27 day incubation at 37°C. The shaded areas represent the fractions that were analyzed by Western Blot. (**B**) Two Western Blots were performed on identical sets of samples. The upper panel detected the presence of αB and the lower panel detected HγD. Numbers along the bottom are SEC fractions. Fractions 13–16 comprised the complex peak; fractions 23–25 comprised the αB peak; fractions 35–37 comprised the I90F peak.

**Figure 6:**
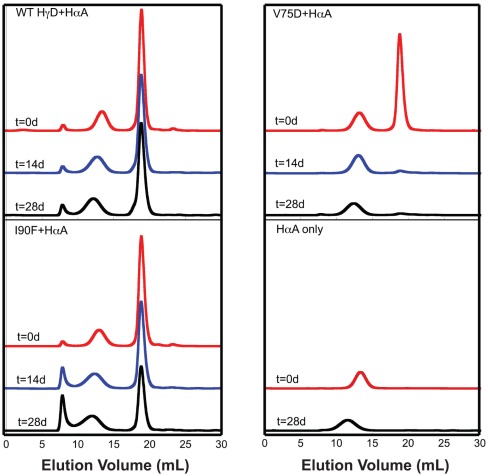
Size exclusion chromatograms of native protein mixtures containing WT or mutant HγD and αA chaperone. Separate samples were prepared for each time point in SEC buffer. Times given are in days. In all cases proteins were present at 1 mg/ml. Each chromatogram is labeled in its upper left corner with the protein mixture.

Results of SEC for samples containing 1∶1 mixtures of γ- and αB-crystallins are shown in Figure 4. For the mixture of WT HγD and αB, no HMW complexes formed between the two proteins. The αB peak remained unchanged over the course of the experiment while the HγD peak broadened slightly. In the case of V75D, the presence of αB did not affect aggregation (Figure 4). Virtually all the soluble protein was lost after 14 days of incubation and aggregates were again visible to the naked eye. HMW complexes between αB and V75D were not present in SEC separations and levels of αB did not decrease, indicating that it was not incorporated into the visible aggregates. Based on these results, V75D appears to aggregate through an intermediate not recognized by αB. This could account for its cataractogenic phenotype in the mouse.

In the case of I90F interacting with αB, after 14 days of incubation at 37°C, samples separated by SEC contained HMW complexes that eluted in the void volume of the column (Figure 4). Individual peaks were still present for both native αB and I90F, and growth of the complex peak in the void volume was observed with concomitant decreases in both of the single protein peaks over time. To verify that this complex peak contained both αB and I90F, Western blots were performed to detect the presence of both αB and HγD. Samples from fractions corresponding to the three major peaks of the I90F + αB mixture (t = 27 days) were analyzed. Four fractions (13–16) corresponding to the complex peak were analyzed (Figure 5A) and fractions 14 and 15 were most abundant in I90F (Figure 5B). This confirmed that the long-lived complex eluting in the void volume contained both α- and γ-crystallin proteins.

### Interactions of αA with Initially Native HγD

In addition to αB, the α-crystallin multimers present in the lens contain a significant proportion of αA. Interactions between the native species were also evaluated for WT and mutant HγD with αA to determine whether recognition patterns differed from those of αB. Experiments were performed in the same manner as described above.

Mixtures containing WT HγD and αA showed some evidence of recognition and complex formation compared to αB. Early-eluting complex peaks appeared in some instances and the major αA peak appeared slightly shifted to an earlier elution time (Figure 6). As in experiments with αB, a significant portion of WT HγD remained soluble throughout the extended incubation times.

Upon mixing V75D with αA, V75D formed large insoluble aggregates that were clearly visible by eye at 14 days. The SEC peak corresponding to soluble V75D decreased in a similar manner as well (Figure 6). Although in a minority of cases a peak was observed in the void after 28 days, which was not the case with αB, αA clearly did not inhibit the large-scale aggregation of V75D, as shown by the loss of the V75D peak at ∼19 ml. Finally, mixtures of I90F with αA displayed similar behavior to those containing αB. A growing peak appeared in the void volume over time (Figure 6). As in the experiments with αB, a significant portion of I90F remained soluble over time.

During the course of the 37°C incubation αA appeared to undergo structural changes that resulted in a larger hydrodynamic radius and earlier elution from the SEC column (Figure 6). Such changes were not found for αB and did not appear to result from the preheating of αA (see Materials and Methods), as protein preparations without heat treatment behaved identically in this respect.

### The Chaperone-bound Conformation of I90F

**Figure 7:**
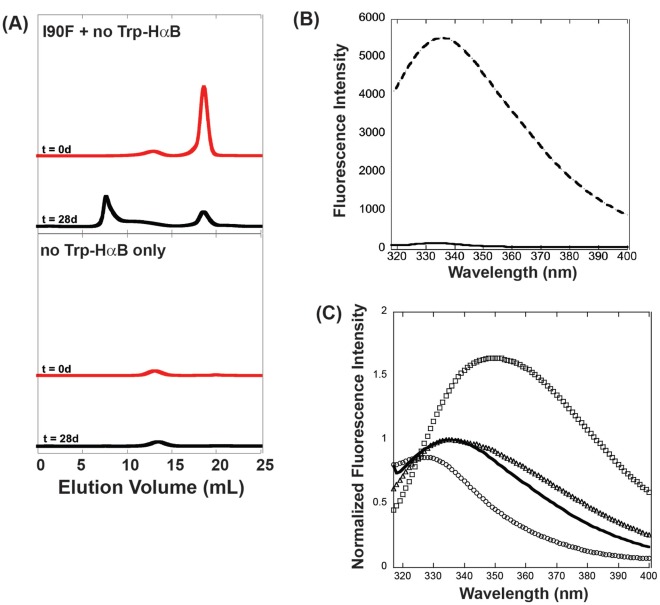
Analysis of native mixing for I90F and W9F/W60F-αB. (**A**) Size exclusion chromatograms of I90F + W9F/W60F-αB mixtures upon initial mixing (0 days) and after the 28-day incubation at 37°C. W9F/W60F-αB alone is shown for comparison. (**B**) Tryptophan fluorescence comparison of WT αB (dashed line) and W9F/W60F-αB (solid line). Proteins were present at 0.05 mg/ml. (**C**) Comparison of tryptophan fluorescence for native I90F (open circles), denatured I90F (open squares), I90F equilibrated in 1.7 M GdnHCl, the transition midpoint of unfolding (open triangles), and I90F in complex with W9F/W60F-αB (solid black line).

To investigate the nature of the interaction between αB and I90F described above, native mixing experiments were performed using αB lacking tryptophans (W9F/W60F) to enable Trp fluorescence measurement of the bound γ-crystallin substrate [22]. W9F/W60F-αB behaved like WT in terms of chaperone activity [22] and did not aggregate over time when incubated under native-like conditions (Figure 7A). Upon initial mixing, there was no interaction between I90F and W9F/W60F-αB and the proteins eluted separately from the SEC column. Following a 28-day incubation at 37°C, protein eluted in the void volume, which corresponded to the complex of W9F/W60F-αB and I90F (Figure 7A). This result is similar to that observed with WT αB and further supports that the mutant W9F/W60F-αB maintains its chaperone activity.

Tryptophan fluorescence was measured for fractions collected from the 28-day samples to determine the general structural state of the bound substrate. In fractions corresponding to the complex peak, fluorescence was significantly higher than that of W9F/W60F-αB in the absence of I90F (Figure 7B). This increased fluorescence must therefore result from bound I90F molecules. However, the fluorescence spectrum corresponds to neither the native nor denatured states of I90F (Figure 7C). Instead, the λ_max_ is most similar to that of partially unfolded I90F, as observed in the transition region of equilibrium unfolding curves [18].

### The Aggregated State of V75D

**Figure 8:**
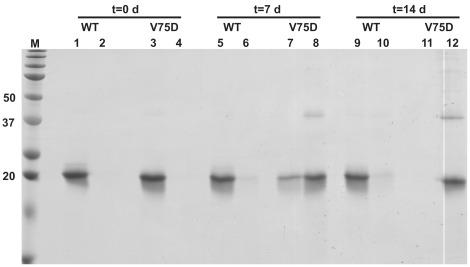
Supernatant (S)/pellet (P) fractionations of WT and V75D incubations at selected time points. Lanes are: **M**, marker with relevant molecular weights noted in kDa; **1**, WT-S t = 0d; **2**, WT-P t = 0d; **3**, V75D-S t = 0d; **4**, V75D-P t = 0d; **5**, WT-S t = 8d; **6**, WT-P t = 8d; **7**, V75D-S t = 7d; **8**, V75D-P t = 7d; **9**, WT-S t = 15d; **10**, WT-P t = 15d; **11**, V75D-S t = 14d; **12**, V75D-P t = 14d. WT samples were analyzed on days 8 and 15, while V75D samples were analyzed on days 7 and 14.

Because V75D aggregated to near completion regardless of the presence of α-crystallin, we set out to determine the nature of the populated species along the aggregation pathway. Pellet/supernatant (P/S) fractionation was used to analyze the insoluble aggregated material (Figure 8). Over time, soluble V75D in the supernatant decreased while aggregated V75D increased in the pellet. While the majority of aggregated V75D was dissociated to the monomer upon treatment with SDS, a distinct dimeric species was present in the pellet fraction on days 7 and 14, as well as small proportions of putative trimeric and tetrameric species on day 14 (Figure 8). These species were resistant to dissociation by SDS + β-ME, suggesting that the dimer is not stabilized by disulfide linkage, or if so, that these linkages are buried and shielded from solvent, even in the presence of SDS. The linkage may be mediated by the aspartic acid introduced by way of mutation, or alternatively, while not directly involved in the covalent chemistry, it may increase the kinetics of a reaction that occurs more slowly in the WT protein. The dimeric species may act as a so-called covalent nucleus for aggregation, in which further addition onto the dimer is non-covalent and thus disrupted in the presence of SDS.

## Discussion

The physiologically relevant disturbances in protein conformation that lead to aggregation of lens βγ-crystallins have been difficult to elucidate. Oxidative damage, such as conversion of glutamines to glutamates, reduces the stability of both the β- and γ- crystallins, but it is not clear that these reductions are sufficient to generate aggregation under native *in vivo* conditions [24]–[26], [41]. The I90F and V75D substitutions in γD-crystallin cause congenital cataracts in mice [11], [49]. However, the mutant proteins fold efficiently within E. coli, and refold *in vitro* under specific conditions, making it unlikely that they represent direct folding defects [18]. Finet and colleagues reported that oxidatively damaged β-crystallins exhibited reduced binding by α-crystallin [41]. We therefore examined more carefully two aggregation pathways as well as their suppression by α-crystallin chaperones.

### Refolding-induced Aggregation Follows the Same Pathway in Both WT and Mutant HγD

For HγD, upon dilution from high concentrations of GdnHCl, the partially folded protein followed an aggregation pathway in kinetic competition with productive refolding. The majority of molecules in solution were incorporated into amorphous aggregates. Both partially folded V75D and I90F chains aggregated to the same extent as WT HγD. Light-scattering curves had the same shape and intensity. This indicated that the reactions proceeded through the same intermediate as for WT and that population of this conformation was not affected by either mutation. If it were, a change in light scattering levels would have been expected. Alternatively, the mutant proteins could have aggregated through a different intermediate, but overall aggregate size and protein incorporation were similar.

Similarly, levels of aggregation suppression by αB were comparable to those observed for WT. This would be expected if the intermediates recognized by αB were the same for WT and both mutants. Because αB recognizes a range of proteins, it is reasonable that it could bind alternative conformations of these γ- crystallin mutants. However, previous results [18], [22] demonstrated that the C-td of both the WT and mutant proteins must be partially unfolded for aggregation to occur, making it likely that the same species was recognized by αB in all cases.

αB effectively suppressed the aggregation of its physiological substrate in experiments where HγD was initially unfolded. The ratio of γD:αB for these experiments was 1∶5, the optimal ratio for suppression determined by Acosta-Sampson and King [22]. The high ratio of αB was required due to the rapid aggregation under these conditions. Lower ratios were successfully used for proteins whose aggregation proceeded at significantly slower rates [52].

Given the duplicated domains of the crystallins, and the presence of intermediates which have an exposed face of a normally buried domain interface, domain swapping is an appealing model for aggregation [53]. Substitutions at the domain interface decreased stability [54]–[56], making exposure more likely. The chaperone may be recognizing the exposed face of one domain, or perhaps an interface between the Greek keys. In another protein deposition disease, light chain amyloidosis, destabilization of the immunogloublin variable domain β-sheet increased amyloidicity, perhaps by a domain swapping mechanism [57], [58].

### Defective Recognition by α-crystallin Suggests Different Mechanisms of Cataract Formation

In contrast to suppression of aggregation competing with refolding, native-like mutant γD-crystallins exhibited altered interactions with α-crystallin chaperones. WT HγD incubated in buffer at 37°C remained highly soluble over weeks, in agreement with the long extrapolated half-time for the unfolding of WT HγD in the absence of denaturant [59]. While HMW complex formation with αA and αB was minimal, some interaction with αA was detected. These interactions could indicate the transient unfolding of a small population of WT molecules, possibly owing to the *in vitro* nature of these experiments.

In contrast to WT, V75D spontaneously aggregated within 7 days and little protein remained in the native monomeric state. This pathway presumably derives from a conformer of the destabilized native-like state, as distinct from the intermediates populated in the refolding protocol. *In vivo* observations by Wang *et al.* showed that nuclear and cytoplasmic aggregates were formed in the mouse lens expressing the murine γD mutant [60].

Neither αA nor αB had a significant effect on the aggregation of V75D. These results are supported by 2-D gel analysis of lens proteins from mice expressing V76D γD. Although the mutant protein was enriched in the water-insoluble fraction of lens protein, levels of soluble α-crystallin were unchanged among WT and hetero- or homozygous mutant lens [60]. The agreement between these results emphasizes that the *in vitro* experiments may serve as suitable models for biochemical analysis of protein stability and protein-protein interactions within the lens, especially considering the lack of lens fiber cell culture.

The substitution could result in local unfolding or could allow hydrophobic core exposure through small changes in backbone conformation. These may not provide recognition sites for the chaperone. An alternative explanation is that the introduction of a charged side chain disrupts a hydrophobic region that would otherwise serve as a binding site for α-crystallin, while the overall intermediate conformation is maintained.

Either case might result in formation of the covalently linked dimer found in V75D aggregates. The linkage may be mediated through the introduced aspartate side chain, or the mutation may increase the rate of formation of the dimer by making other reactive groups more accessible. Overall, this suggests that in the lens, the aggregation-prone species may evade sequestration by α- crystallin and form light-scattering aggregates.

When incubated alone, native I90F gradually accumulated aggregated material over the 28-day period. In the presence of either αA or αB, I90F formed a HMW complex with the chaperone whose population increased over time. The slow decrease of I90F monomers with time, in opposition to V75D, suggests the gradual unfolding and population of an aggregation-prone species. This makes I90F an ideal target for sequestration by the components of α-crystallin.

Other studies have confirmed that α-crystallin is a better chaperone of slower aggregation processes [37], [61] supporting this interpretation. A similar interaction with bovine α-crystallin was observed for I4F murine γB-crystallin in *in vitro* mixing experiments at elevated temperatures [62]. In lens extracts from mice harboring this mutation, a complex was formed between α- crystallin and γ- crystallins, presumably the I4F mutant [62].

While analogous behavior *in vivo* would prevent the growth of large aggregates of I90F, the finite supply of α-crystallin could become saturated with mutant protein molecules more quickly than in the normal lens. This would impede further chaperone activity and likely compromise other interactions, such as those with lenticular cytoskeletal proteins [42], [63], [64].

Recent findings by Mchaourab and colleagues complement those for V75D described here. In particular, they observed that under native conditions, the protein does not interact with αA or αB and only in the case of a highly destabilized double mutant does the chaperone recognize its substrate [50]. However, they did not observe appreciable formation of HMW complexes between α-crystallin and the substrates investigated [50].

In essence, our results are in agreement with the model proposed by Mishra *et al* [50] in that unfolding of the C-td triggers recognition and suppression of aggregation by the chaperone. V75D, though significantly destabilized in the N-td, populates a conformation whose aggregation is not suppressed presumably because its C-td is not sufficiently denatured. The HMW complexes that elute in the void volume correspond to chaperone-bound substrate with compromised C-td stability and/or structure. This conclusion is also supported by studies on the fluorescence properties of bound substrates by Acosta-Sampson and King [22].

Point mutations in HγD resulting in single amino acid substitutions lead to very different causes of cataract. The well characterized P23T and R58H substitutions dramatically reduce protein solubility [14], [16], [51], [65], [66]. R14C results in disulfide-mediated aggregation [67] and R36S increases the propensity for crystallization [13], [14]. The work presented here expands on how the destabilizing mutations V75D and I90F may result in cataract disease. In particular, these results support the proposal that multiple mechanisms may lead to cataract formation and these biochemical analyses can provide initial models of *in vivo* events.

## Materials and Methods

### Cloning, Protein Expression and Purification

WT HγD and mutant proteins V75D and I90F were prepared as previously described [18]. Both WT and W9F/W60F-αB were expressed and purified as previously described [22]. WT αA was expressed similarly and purified following procedures modified from [52]. Two rounds of anion exchange chromatography were performed. In the first round, αA was pooled from the sample flow-through. This was re-applied to the column and eluted in a step gradient of 10%, 25%, and 100% B (Buffer A: 50 mM Tris, pH 8.0; Buffer B: 50 mM Tris, 1 M NaCl, pH 8.0). Ion exchange was followed by size exclusion chromatography in 50 mM sodium phosphate, 150 mM NaCl, pH 7.0, using a Superose 6 10/300 GL column (GE Healthcare, Piscataway, NJ). Protein concentrations were determined by UV absorbance at 280 nm using the following extinction coefficients: 42,860 M^−1^ cm^−1^ (WT, V75D and I90F HγD), 14,440 M^−1^ cm^−1^ (αA) and 13,980 M^−1^ cm^−1^ (αB). Extinction coefficients were calculated using ExPASy ProtParam [68]. The concentration of W9F/W60F-αB was measured using the BCA assay (Pierce, Rockford, IL).

### Aggregation and Suppression of Aggregation

Assays were based on the protocols of Acosta-Sampson and King [22]. WT and mutant HγD proteins at 1 mg/ml were unfolded by incubating overnight at 37°C in 5 M GdnHCl, 100 mM sodium phosphate, 1 mM EDTA, 5 mM DTT, pH 7.0. Unfolded protein was placed in a quartz cuvette and diluted 10-fold with refolding buffer (100 mM sodium phosphate, 1 mM EDTA, 5 mM DTT, pH 7.0) to achieve final concentrations of 0.1 mg/ml HγD and 0.5 M GdnHCl. Samples were mixed by rapidly pipetting upon addition of buffer. Solution turbidity (A350) was measured continuously for 20 minutes, beginning immediately after sample mixing. Aggregation suppression assays were performed in the same manner, with the addition of αB in the refolding buffer at a final concentration of 0.5 mg/ml. Cuvette temperature was maintained at 37°C using a single cell Peltier controller and all protein and buffer solutions were maintained at 37°C during the experiments. Experiments with each protein were performed at least in triplicate.

### Native Interaction Assays

Each of the HγD proteins, WT, V75D and I90F, were mixed with either αA or αB in a 1∶1 ratio at concentrations of 1 mg/ml in SEC buffer. For samples containing αA, the chaperone was preheated at 42°C for 15 minutes prior to sample preparation. Samples were then incubated in a 37°C warm room with constant gentle rotation for up to 28 days. At various time points, samples were removed, filtered through a 0.2 µm membrane, and applied to a Superose 6 10/300 GL column. Fractions were collected every 0.5 ml and SDS-PAGE samples were reduced and boiled immediately following separation for further analysis. Fractions were assessed for formation of α:γ complexes, as well as for changes in free α and free γ peaks. Control samples were prepared containing either αA or αB only, or the individual HγD proteins, each at 1 mg/ml, and treated identically to experimental mixtures. Experimental mixing samples were prepared and analyzed at least in triplicate and controls were prepared and analyzed in duplicate or triplicate.

0.5 ml fractions collected from native interaction sample separations were electrophoresed through 14% SDS-PAGE gels and proteins were transferred to 0.2 µm pore size PVDF membranes (Millipore, Billerica, MA). Sets of identical membranes were probed with primary antibodies for αB and HγD (Santa Cruz Biotechnology, Santa Cruz, CA). Alkaline phosphatase-conjugated secondary antibodies were used in conjunction with the Immun-Blot colorimetric assay (Bio-Rad, Hercules, CA) for signal detection.

Identical native mixing assays were performed using W9F/W60F-αB and I90F HγD. Samples were filtered and applied to a Superose 6 10/300 GL column at 0 and 28 days post-mixing. 0.5 ml fractions were collected. The Trp fluorescence was measured for the fractions corresponding to the αB:γD complex, free αB and free HγD to determine the conformation of I90F when bound by αB. Measurements were taken with a Hitachi F - 4500 fluorescence spectrophotometer using the following parameters: λ_ex_ = 300 nm; λ_em_ = 310–400 nm; excitation and emission bandwidths  = 10 nm; scan rate  = 60 nm/min.

P/S separations were used to analyze the partitioning of V75D protein over time. Samples containing 1 mg/ml V75D (native mixing control samples) were incubated as described above. After 0, 7–8 and 14–15 days aggregates were pelleted by centrifugation at 10,000×*g* for 20 minutes at 4°C. Supernatants were carefully removed, reduced and boiled with SDS sample buffer. The pellets were washed twice with SEC buffer. Pelleted material was resolubilized by boiling in sample buffer containing 2% SDS and β-ME. Samples were electrophoresed through 14% polyacrylamide gels and Coomassie stained. WT HγD incubated over the same time period was used as a control and treated identically.
